# Rivaroxaban After Transcatheter Aortic Valve Replacement: A Critical Appraisal of the GALILEO Trial

**DOI:** 10.1007/s10557-022-07350-y

**Published:** 2022-06-14

**Authors:** Patrick Sulzgruber, Andreas Hammer, Alexander Niessner

**Affiliations:** Division of Cardiology, Department of Internal Medicine II, Medical University of Vienna, Waehringer Guertel 18-20, 1090 Vienna, Austria

**Keywords:** TAVR, Rivaroxaban, NOAC, GALILEO trial

## Abstract

**Purpose:**

The anti-thrombotic approach in individuals undergoing transcatheter aortic valve replacement (TAVR) mirrors a controversial field in clinical practice.

**Methods/Results:**

The aim of this article was to critically appraise the randomized controlled GALILEO trial, where two different antithrombotic regimes (10 mg rivaroxaban + 3 months aspirin vs. aspirin + 3 months clopidogrel) were compared in patients who underwent TAVR as well as available evidence in literature in this field.

**Conclusion:**

The GALILEO trial was prematurely terminated as a consequence of increased risk of both death or thromboembolic complications and a higher risk of bleeding in the anticoagulation arm, compared to the antiplatelet-based strategy.

Various concerns have been raised that the negative results of the GALILEO trial need to be regarded with caution. A routine use of oral anticoagulation (OAC) for the prevention of atherothrombotic events and valve thrombosis after TAVR in individuals who do not have an indication for oral anticoagulation, can currently not be recommended when considering the evidence base of available literature. However, the negative results of the GALILEO trial need to be interpreted with caution – especially in terms of dose of rivaroxaban – and should not discourage from performing further trials investigating safety and efficacy of this therapeutic approach. Additionally, further dose-finding trials for rivaroxaban should be considered.

Adverse thromboembolic events – such as ischemic stroke, valve thrombosis or systemic embolism – are frequently observed at an early stage after transcatheter aortic valve replacement (TAVR) in a population at high risk for cardiovascular events. Several observational data suggest that subclinical leaflet thrombosis on the implanted bioprosthetic valve is a major driver for the respective adverse events. While current ESC guidelines recommend a dual anti-platelet therapy (DAPT) after TAVR with a level of evidence C and class IIa of recommendation, the optimal anti-thrombotic treatment strategy after this valve intervention remains unclear and continues to be the object of current discussions [1]. Considering the thrombotic risk after TAVR, the initiation of oral anticoagulation (OAC) potentially lowers the risk of thromboembolic events but possesses an inherently elevated bleeding risk. However, there is no available evidence for the routine use of anticoagulation after TAVR in terms of efficacy and safety.

Current available guidelines coherently favor DAPT, but it should be taken into account that these recommendations are primarily based on expert consensus and extrapolation data of related interventions and surgical procedures. The respective sub-group analysis of ENGAGE AF-TIMI 48 and ARISTOTLE investigated the safety of NOACs in individuals that underwent biological aortic valve replacement.

Addressing the safety and efficacy of a single OAC approach after TAVR, the data of the POPular-TAVI trials’ B cohort, where sole OAC was compared to OAC + clopidogrel in TAVR patients with an indication for OAC, emphasized the superiority of single OAC therapy compared to OAC in addition to 3 months of clopidogrel in terms of bleeding events [2].

These results remained stable within a subgroup analysis for NOAC [2]. However, profound data on the optimal antithrombotic regimen using NOAC agents in terms of efficacy and safety after TAVR remain inconclusive.

Considering this gap of knowledge, the randomized controlled GALILEO trial aimed to investigate the impact of an OAC strategy with rivaroxaban at a dose of 10 mg daily (including 3 months of concomitant aspirin) as compared with an antiplatelet strategy (aspirin indefinitely coupled with 90 days of clopidogrel) in patients without established indications for anticoagulation after successful TAVR. It was observed that a postoperative rivaroxaban-based strategy was associated with increased ischemic and bleeding events in comparison to an antiplatelet therapy approach [3].

However, the study was prematurely terminated as a consequence of increased fatal events in the rivaroxaban group. The primary efficacy outcome was defined as all-cause death, stroke, myocardial infection, pulmonary embolism, symptomatic valve and deep-vein thrombosis which was observed in 9.5% of the standard care group and 12.7% in the rivaroxaban arm (*p* = 0.04).

Of note, only 183 of 440 estimated primary efficacy outcomes occurred within the observation period, resulting in a statistical power of only 54%. To achieve the initial planned 80% power in compliance with the observed primary efficacy event rates and 18 months of follow-up, the study cohort had to be quadrupled in size. All this fosters the assumption of a substantial mismatch between estimated and observed primary efficacy events.

On the other hand, primary safety outcome consisted of major, disabling, or even life-threatening bleeding events which appeared in 3.8% patients of the antiplatelet and in 5.6% of the rivaroxaban group (*p* = 0.08). Notably, non-cardiovascular causes of death deviated even further between the two groups, with 3.5% for the rivaroxaban and 1.3% for the antiplatelet strategy, respectively. However, the primary efficacy outcome occurred more rarely than it was assumed by the initial sample size calculation, which highlighted that a study population of at least 6572 individuals would have been needed to draw a certain conclusion on the primary outcome rates.

Considering GALILEOs’ study protocol, there is no well-grounded rational for the applied 10 mg once-daily dosage of rivaroxaban and it remains unknown why the investigators chose an addition to aspirin and not a sole OAC approach. The authors refer to the RECORD trial using the respective dosage for the prevention of venous thromboembolism after major orthopedic surgery [4]. Both the phase-II ATLAS ACS-TIMI46 and the phase-III ATLAS ACS 2-TIMI51 trials highlighted a directionally favorable net clinical outcome – in terms of death, myocardial infarction, stroke, or TIMI major bleeding – in participants receiving the 2.5 mg or 5 mg twice-daily dosing in addition to standard aspirin as compared to dual anti-platelet therapy [5, 6]. ATLAS ACS-TIMI46 investigated dose-finding of rivaroxaban in patients after acute coronary syndrome (ACS) and concluded that the administration of 2.5 or 5 mg rivaroxaban provided a favorable net clinical benefit in a twice daily obtained regimen to further promote drug level stability and may lead to a reduction in major ischemic events [5]. Subsequently, the ATLAS ACS 2-TIMI51 aimed to establish a clinically effective low-dose rivaroxaban treatment regimen and highlighted that rivaroxaban was associated with reduced risk for cardiovascular death, myocardial infarction, and thromboembolic events in ACS patients [6]. Furthermore, the regimen of 2.5 mg twice daily showed no elevated risk for fatal bleeding events and reduced both cardiovascular and overall mortality [6]. Additionally, the PIONEER AF-PCI trial reported positive results with similar rivaroxaban dosing in individuals with AF that underwent percutaneous coronary intervention (PCI) comparing 15 mg rivaroxaban once daily, 2.5 mg rivaroxaban twice daily both in addition to a P2Y12 inhibitor, compared to VKA and DAPT [7]. Both rivaroxaban groups were associated with lower bleeding risk compared to the triple anti-thrombotic therapy with VKA and DAPT treatment [7]. Considering the available evidence in literature on the varying dosage of rivaroxaban in addition to anti-platelet agents, the rationale for the regimen used in GALILEO seems unclear. Alternatively, a higher dosage of rivaroxaban without aspirin might provide a net clinical benefit as shown by the AFIRE study [8]. Within this trial 15 mg (or 10 mg if GFR ≤50 ml) of solitary rivaroxaban was compared to rivaroxaban and concomitant aspirin (or P2Y12 inhibitor) in individuals with stable CAD and AF in an Asian population. The rivaroxaban monotherapy was associated with decreased risk for major bleeding events and furthermore showed noninferior results in terms of the prevention of cardiovascular events and all cause death. Therefore, the omission of aspirin promoted a superior net benefit. In this regard, results of the POPular-TAVI trials’ B cohort highlighted a superiority of sole OAC in terms of bleeding risk, but noninferiority for prevention of cardiovascular death and thromboembolism [2]. Hence, available evidence supports a single OAC agent approach in TAVR patients due to its association with decreased bleeding events. In addition, 1 year follow-up data of the POPular-TAVIs A cohort, where solitary aspirin was compared with standard care DAPT (aspirin indefinitely + 90days clopidogrel), emphasized the redundancy of clopidogrel in TAVR patients without OAC indication. Sole aspirin showed superior safety outcome while being noninferior in efficacy [9].

Comparing those results with the data gathered by the GALILEO trial fosters the assumption that aspirin was presumably redundant and might have contributed to excessive bleeding events in the rivaroxaban arm.

Conversely, patients started to appear undertreated beyond the 45 day of the observation period, where combined therapy with rivaroxaban and aspirin was still established. This might highlight the underlying cause of inappropriate rivaroxaban dosage, whose efficacy has not yet been tested in a comparable patient population.

Moreover, the GALILEO-4D sub-study investigated the impact of these two different antithrombotic approaches in relation to valvular leaflet thickening and dysfunction of the prosthetic valve. Both subgroups were evaluated and screened via contrast-enhanced four-dimensional CT 90±15 days after randomization [10]. In contrast to the main trial, the rivaroxaban group showed superior outcome in terms of lower reduced leaflet motion (2.1% vs. 10.8%) and thickening (12.4% vs. 32.4%) compared to the fraction, which received only antiplatelet agents. Thus, rivaroxaban appeared superior in preventing subclinical leaflet degradation. However, the beneficial effect of OAC on valve deterioration, as shown in the GALILEO-4D sub-study was only investigated the first 3 months where aspirin was still prescribed. Thus, it remains unknown to what extent the antiplatelet agent contributed to this result, and moreover, to the course after discontinuation of aspirin.

## Conclusion and Future Perspective

A routine use of OAC for the prevention of atherothrombotic events and valve thrombosis after TAVR in individuals who do not have an indication for oral anticoagulation can currently not be recommended when considering the evidence base of available literature. However, the negative results of the GALILEO trial need to be interpreted with caution – especially in terms of the dose of rivaroxaban – and should not discourage from performing further trials investigating the safety and efficacy of this therapeutic approach. Additionally, further dose-finding trials for rivaroxaban should be considered. Data of ongoing trials, including ENVISAGE-TAVI AF and AVATAR, might allow drawing a certain conclusion on the net clinical benefit of this antithrombotic treatment approach in individuals undergoing TAVR.

## Data Availability

Not applicable
